# Superior Conductivity of Transparent ZnO/MoS_2_ Composite Films for Optoelectronic and Solar Cell Applications

**DOI:** 10.3390/gels9110906

**Published:** 2023-11-16

**Authors:** Shahad Tareq, Gokhan Kirkil, Bengü Özuğur Uysal

**Affiliations:** Faculty of Engineering and Natural Sciences, Kadir Has University, Cibali, Fatih, 34083 Istanbul, Turkey

**Keywords:** sandwich-structured thin films, transparent conducting oxide, ZnO/MoS_2_ composite, optical and electrical properties

## Abstract

The use of transparent conductive oxides in optoelectronics created a revolution where new-generation materials with high transmittance, low sheet resistance values, durability, and portability can be achieved without decreasing efficiency or increasing costs. Transparent ZnO/MoS_2_ sandwich-structured conductive composite films were produced in this study via the sol–gel method, which is considered the most efficient method due to its simple process and low cost. The crystal structure properties of ZnO/MoS_2_ were characterized via X-ray diffraction (XRD) patterns. The crystal sizes of ZnO films doped with different amounts of MoS_2_ were determined. A UV–visible absorption spectrometer was used to perform the spectroscopic analysis of the film. The area under the absorption curve and the full width of the half-maxima of absorbance data were calculated. Using these values, the optimum amount of MoS_2_ was determined for the best additive distribution. In addition, in order to determine the best transparent conductive material, resistance values measured via the four-point probe method were compared for different MoS_2_ additive amounts. The optical and electrical characterizations of transparent ZnO/MoS_2_ conductive oxide films were investigated. According to the parameters obtained via UV–vis spectroscopy, XRD, and four-point probe measurements, the most effective dispersion that exhibits a low width ratio and high resonance ratio was found for ZnO/MoS_2_ with a doping amount of 4 mg, the crystallite size of the films was found to be within the range of 21.5 and 24.6 nm, and these observations demonstrated a figure-of-merit value of more than 4.8 × 10^−2^ with respect to these sandwich-structured films. Compared to the values of previous studies on various transparent ZnO-doped conductive oxide materials, it is possible to claim that these new films have a structure that is very similar to the transparent conductivity characteristics of other films, and they may even be superior relative to some MoS_2_ amounts.

## 1. Introduction

Transparent conductive films are mainly composed of materials called transparent conducting oxides (TCOs), as the name suggests, and they are distinguished by their ability to be optically transparent and their high electrical conductivity. Since transparent conductive oxide films must both be transparent and conductive, the figure-of-merit (FoM) value, which expresses the effect of both permeability and conductivity values, can be calculated. Based on these properties, TCO thin films are used in applications and solar cells. In addition, TCOs have an important role in various applications, such as flat-screen panels, photocatalysts, solar cells, batteries, and computing devices. The material in the form of a film consists of layers that are deposited on top of each other at a certain wavelength. It acts as a resistor against the expulsion of charge carriers from the photocell, which in turn acts as a window allowing light rays to pass into the layers below. The energy difference response of the cells is less than the visible wavelength from 380 nm to 750 nm.

Photons outside this band gap run out, which means that visible light passes through it. However, photovoltaic applications need a wide band gap to prevent unwanted absorption of the spectrum. The manufacture of thin films has a broad variety of uses, as its architecture starts from a basic single coating to complicated configurations that comprise a hundred or more layers, which are called multiple dielectric coatings. These applications involve the manufacture of optical modules [1], which greatly decrease the surface reflectivity of the optical object, such as polarizers, interference splitters, and antireflection coatings [2]. In recent years, TCOs have been essential in optoelectronics applications due to their unique properties, such as good conductivity, high transparency in the visible region, reflectivity for IR beams, and poor light absorption materials [3]. The effectiveness of these deposition techniques, which include chemical vapors, pulsed laser deposition, sputtering, spray pyrolysis, evaporation, and the sol–gel dip/spin-coating method, must be considered to maintain TCO thin film performances.

Currently, the most preferred TCO for optoelectronic applications is indium-doped tin oxide (ITO). ITO, known as the best transparent conductive material, contains indium additives, an enemy of living organisms; its mechanical instability and difficult production process limit the applications of TCO materials [4]. Sputtering, pulsed laser depositions, chemical vapor deposition, and chemical spray are used in TCO fabrication. To manufacture SnO_2_, some elements are used as dopants. Dawood et al. showed that the propagation of optics is highly related to doping in the thin films of indium tin oxides (ITO) that are related to free electrons. They observed that ITO thin films frequently improve optical transmission with respect to doping levels as conductivity increases. With the preparation of dense indium-containing ITOs, light transmittance decreased by about 2%. By changing the contribution percentage, it is possible to significantly improve transmissions. The reduction in transmission may be induced by the improved dispersion of photons via the crystal defects produced by doping. Due to the impact of transmissions on doping concentrations and deposition, other TCO output properties are often affected [5]. Jafan and coworkers used the sol–gel process to manufacture ITO thin films, with polyvinyl alcohol as a polymerizing agent and binding material. Low resistivity (sheet resistance of 2.5 kΩ/cm^2^) and highly transparent films were obtained and can be applied as covers on heater windows [6]. ITOs, which constitute the main material of television screens and all touch screens, contain indium; the damage caused by the degradation of indium threatens the health of living things.

Despite its convenient optical and electronic properties, research with respect to alternative materials with features that can replace ITO intensified. Various alternatives were proposed for overcoming these limitations, and plentiful inexpensive materials attracted substantial attention. Among these various materials, zinc oxide (ZnO) has emerged as one of the most promising. ZnO exhibits high optical transparency within the visible region but low electrical conductivity. In order to improve the electrical properties of ZnO without impairing its unique optical properties, doping with group III elements (In, Al, and Ga) was attempted [7]. The transparent conductive oxide of Al–ZnO characterizes its high transmittance and low resistance in visible regions. It is possible to control the bandgap by doping with Al. ZnO was used in applications that require high conductivity and low resistivity, such as solar cells and optical coatings. Lu et al. found that the optical absorption spectrum evaluation of E_g op_ was primarily related to the intensity of carriers; thus, it is essential to the aluminum content [8]. Burstein-Moss [9,10] reported that when *n_e_* ≤ 4.2 × 10^19^ cm^−3^, the optical gap increases with respect to electron concentrations; unexpectedly, a decrease in the energy gap occurs at 5.4–8.4 × 10^19^ cm^−3^, which is compatible with the Mott SMT criteria [8]. Park et al. and Agura et al. found that on low resistivity, two types of films were prepared by using PLD: GZO and AZO. ZnO thin films exhibit good resistance between 5 and 10 cm; moreover, ZnO thin films that were prepared using PLD exhibit the best conductivity in comparison to other processes. Due to high costs and stability problems, the wide application of this process was eliminated [11,12]. The high FoM of indium zinc oxide (IZO) films deposited at room temperatures comparable to those of ITO shows that they are promising for applications in OLEDs [13], organic solar cells [14], and flexible electronics [15]. As a semiconductor, ZnO is known for having fast transparent prime properties at room temperature and being efficient relative to light harvesting used in solar cell applications; moreover, it provides good thermal and mechanical stability. Commonly, the thin films of undoped ZnO usually show n-type conduction. Generally, semiconductors have a wide bandgap and high electron–hole recombination; a noble metal, such as Au and Ag, and nanoparticles such as AuPd are added to obtain a narrower bandgap [16,17]. ZnO has a broad range of excitons, strong binding force, high strength, high stiffness, and larger specific electron mobility. For these reasons, indium-doped zinc oxide (IZO) films seem to be an alternative to ITO films. However, recycling indium-containing IZO films is not possible due to the damage it inflicts on health. Due to increasing costs in recent years because of a decrease in indium resources, another element or compound that can provide the same level of conductivity should be doped relative to zinc oxide (ZnO). Thin films of Ga-doped ZnO (GZO) were prepared by Shin et al. using RF magnetron sputtering on glass and Al_2_O_3_ (0001) [18]. They found that there are visible grains in substrates with the same process condition relative to GZO. In the GZO film on the Al_2_O_3_ substrate, however, distinguishing individual grains is difficult. Compared with epitaxially grown GZO films on Al_2_O_3_ substrates, the superior crystal content of epitaxially grown GZO films is observed. Improved electrical characteristics can be attained using polycrystalline GZO films on glass substrates. The film’s crystallinity, stoichiometry, and the resistivity and transmittance curves of Si-doped ZnO thin films deposited on both glass and PET substrates were calculated by Clatot et al. Their findings have verified that electrical and optical properties are highly dependent on the existence of the substrate. With respect to the hydroxylation of surface vacancies, interstices are studied theoretically and experimentally. Extrinsic doping and other electronic properties are studied for ZnO surfaces [19]. Kim et al., in another study of the sol–gel spin-coating process, deposited thin films of Al-coated zinc oxide on quartz substrates using the sol–gel spin-coating technique. As a starting material, zinc acetate dihydrates, Zn (CH_3_COO)_2_·2H_2_O were used as the stabilizer, and solvents comprised monoethanolamine, C_2_H_7_NO and 2-methoxyethano, CH_3_OCH_2_CH_2_OH. Aluminum nitrate, Al (NO_3_)_2_·9H_2_O was the dopant source. AZO-thin films were heat-treated for 60 min at 550 °C [20]. With respect to another method of obtaining metal oxide-doped ZnO films, the principle of the process is based on a nonaqueous dip-coated sol–gel method using a microwave. However, this process results in high resistivity; by improving each step in the sol–gel process—including the production of nanoparticles, dispersion, thin film treatment, and annealing—the production of thin films can improve [21].

The new doping material relative to ZnO, which will act as a proxy for indium, must be capable of responding to the multifunctional demands of today’s ever-changing technology. The annealing effects of CuS, CuZnS, and ZnS thin films on structural, electrical, and optical properties were investigated by Yildirim et al., and they reported that annealing seems to be the most suitable thermal process for manufacturing TCO thin films. Because an increase in annealing temperatures imparts higher resistance on annealing films than grown films, this also minimizes the optical band gap. Instead of strengthening the crystals, hardening and tempering will further disorganize the crystals. Annealing involves heating a material beyond its recrystallization temperature, maintaining an acceptable temperature so that it can be converted into a sample in time, and then gradually refreezing the sample. During the annealing process, atoms pass through the crystal structure and reduce dislocations, which are caused by several changes in the softness and hardness of the crystal. The volume of crystallinity or the particles of the thin film do not change when applying thermal annealing. The amount of contextual oxygen is reduced to increase conductivity. Moreover, an increase in annealing processes and the temperature of heat treatment atmospheric reduction increases conductivity. By reducing oxygen interstices, an increase in conductivity is achieved. In this manner, physical contact enhancements increase conductivity when annealing temperatures increase at high rates [22]. On the other hand, another material that manifests excellent physical and chemical properties is two-dimensional MoS_2_. It has a large surface-to-volume ratio; a significant band gap approximately starts from 1.2 eV, and its maximum energy is 2.2 eV; also, it has many active edges and its electrons exhibit high mobility within the plane. By regulating film thicknesses, MoS_2_ can realize tunable electronic/photoelectron performance. Furthermore, MoS_2_ has strong chemical stability, and combining and utilizing silicon is also not difficult with respect to the processes in CMOS logic products. Short channel effects can effectively be suppressed by ultrathin MoS_2_ nanosheets. The nanomaterial components of MoS_2_ are used in many applications because of these advantages, such as the batteries of electronic/optoelectronic devices, photocatalysis, lithium-ion devices, and lithium-ion nanomaterials. However, only 5.6 percent of the bulk content is absorbed by the monolayer’s MoS_2_, which significantly restricts its use in photodetector (PD) devices and photocatalysis. Nano-molybdenum disulfide is commonly combined with other near-atomic-distance semiconductors to create a composite that can satisfy these applications. Therefore, MoS_2_ can be considered as an alternative additive to indium relative to ZnO doping, and it can provide both high conductivity and transparency.

The specific objectives of this study were to study the electrical and optical characteristics of transparent ZnO/MoS_2_ conductive thin films. However, when preparing this new TCO film, an important consideration is to distribute the additive material homogeneously in the main material, similarly to other methods. When there is a small amount of MoS_2_ additives (low molar ratio), the conductivity mechanism cannot be produced because, in a very sparse structure, conductive electrons cannot be transmitted from end to end. Due to possible discontinuity in the carrier conduction line, the density of the additive should be optimized. On the other hand, by adding excess MoS_2_ to ZnO, it is possible to make the composite material more conductive. Therefore, it is crucial to determine the following: what technique to use, the stage of the process at which to apply the technique, and how much MoS_2_ should be added. In order to obtain a homogeneous material with the desired properties, it is necessary to mix the starting chemical solutions in certain proportions and repeatedly check whether the particles belonging to different chemicals are homogeneous in the material. These trials should continue until the best value is found. There are many methods for determining the distribution of particles: SEM, TEM, and light scattering (zeta sizer). It is impossible to measure all materials prepared at different concentrations with these methods as they can be expensive. Optical methods, which are extremely reliable, easy, and cost-effective, one can calculate the resonance ratio and normalized width values from the absorption response of the composites according to wavelength. Then, the dispersion rate of MoS_2_ in the composite film is determined. A proper ultrasonication process has been realized to maintain a good dispersion of the MoS_2_ inside the ZnO matrix, lowering the normalized width and increasing the resonance ratio.

## 2. Results and Discussion

### 2.1. Optical Properties of ZnO/MoS_2_ Composite Films and Dispersion Calculations

**Figure 1:**
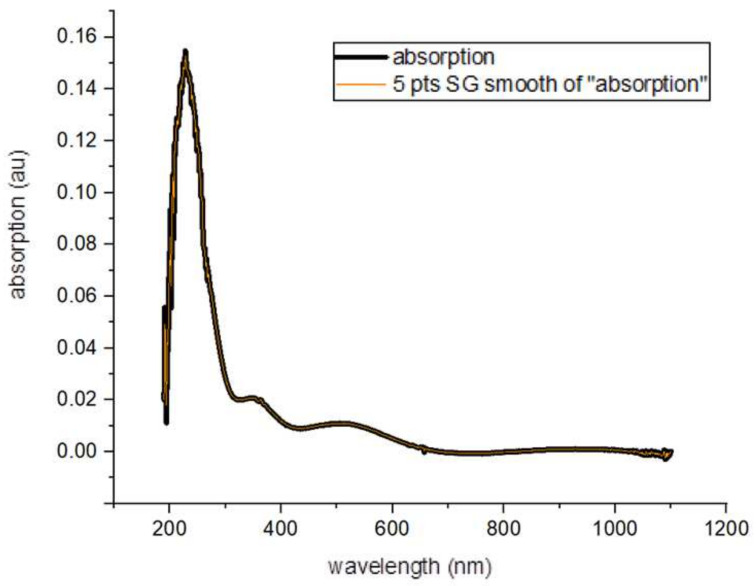
The absorption curve of the composite after smoothing.

**Figure 2:**
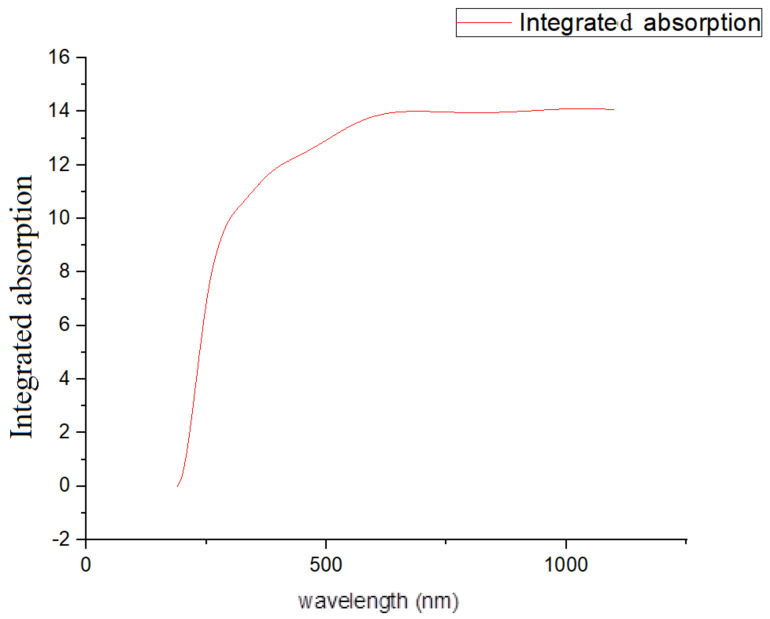
The integration of the absorption–wavelength curve yields the resonance area of absorption.

**Figure 3:**
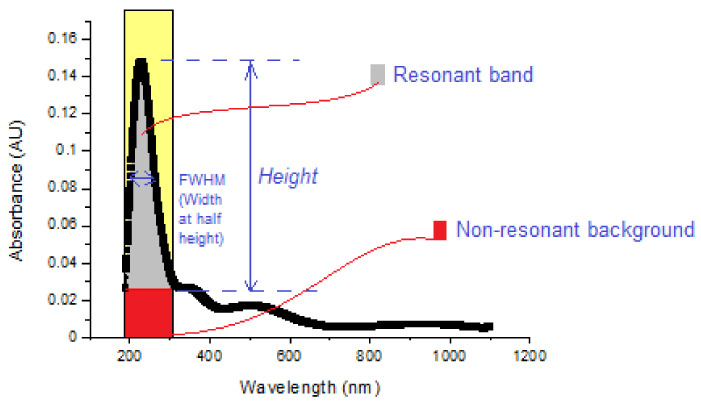
The definition scheme of the resonant band, nonresonant background, and width and height of the resonant band in order to calculate the resonance ratio and the normalized width of dispersion [23,24].

First, the smoothing of the curve was realized using Origin 8.0 (Figure 1). Afterwards, the area of the resonant band was calculated using the integration property in Origin 8.0 (Figure 2). When calculating the nonresonant background area, the maximum part of the absorbance curve was used. The area of that part then needed to be calculated. For obtaining values on either side of the maximum curve, the wavelength values corresponding to the absorbance value were taken. The part of the curve with these values formed a line. Then, the equation for this line was obtained, and the area under this line was calculated. If the area below the line (the nonresonant background) is subtracted from the area under the maximum curve, the area of the resonant band is obtained (Figure 3):

Resonance ratio = (area of resonant band)/(area of nonresonant background)(1)

Also, the normalized width at half the peak’s height can be found by taking the ratio of the width of the resonant band to the height of the resonant band. The normalized width of the absorbance curve is determined via the following formula:

Normalized width = (width of resonant band)/(height of resonant band)(2)

The most effective dispersion exhibits a low normalized width and high resonance ratio [23].

**Figure 4:**
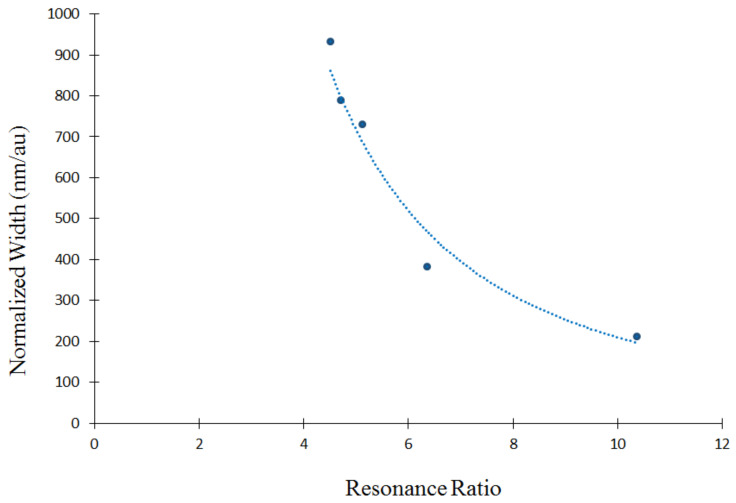
The normalized width versus resonance ratio curve to determine the best-dispersed film.

The importance of dispersion renders these properties intensive properties, which are not changed relative to road path/wavelength. The use of different concentrations shows different effects on dispersion factors, which shows a decrease in the peak’s width with an increase in sonication time, which means that the resonance sheet becomes sharper and higher and therefore results in an increase in nanoparticles. Table 1 summarizes the calculations using dispersion theory. As observed in Table 1, an increase in the doping amount results in an increase in the area of the resonant band until the amount of 4 mg. In addition, the resonance ratio of the samples decreases; afterwards, it increases with an increase in the doping amount. The best dispersion can be obtained for the lowest normalized width and the highest resonance ratio. To obtain the best dispersion, new figures must be plotted for these values. Figure 4 illustrates the normalized width versus the resonance ratio curve. The most effective dispersion that exhibits a low width ratio and high resonance ratio was observed relative to the doping amount of 4 mg.

**Figure 5:**
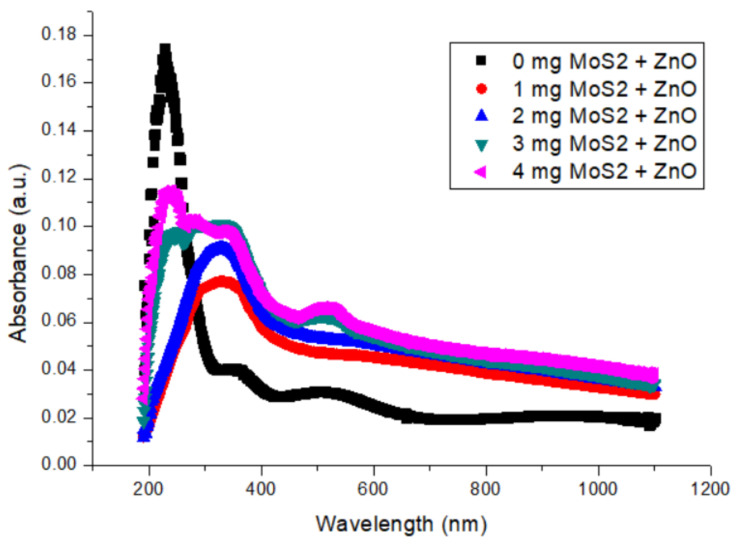
The absorption response of ZnO films with various MoS_2_ doping amounts.

Figure 5 illustrates the normalized absorption curves of ZnO films with various MoS_2_ doping amounts. The absorption response of the nondoping film exhibits the same ZnO characteristic peak near 240 nm, as observed in Figure 5. This peak shifts to longer wavelengths with an increase in the doping amount of MoS_2_ because MoS_2_ has a characteristic peak at another wavelength (around 500 nm), which is longer than the peak of ZnO. The shift becomes larger relative to an increase in doping amounts. Finally, it is obvious that there is more than one peak in the absorption response of composite films. The compared absorption intensities show that an increase in MoS_2_ doping amounts promotes opacity, which leads to an increase in absorbance intensity as expected. This is proof of well-dispersed composite films.

**Figure 6:**
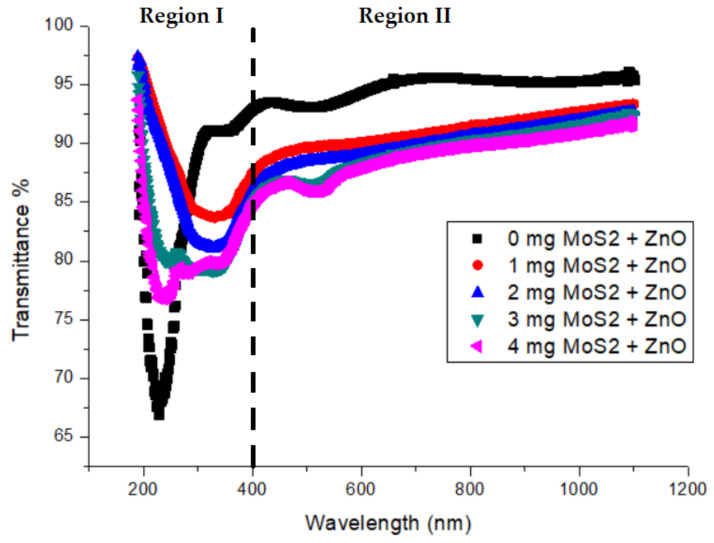
The transmittance of ZnO films with various MoS_2_ doping amounts.

Figure 6 depicts the transmittance graphs of ZnO films with various MoS_2_ doping amounts. It is obvious that the transmittance values of the films decrease relative to MoS_2_ doping amounts. The graphs can be divided into two regions: a short-wavelength region (Region I) and a long-wavelength region (Region II). The optical properties of the composite material differ in these two regions. It appears that the opacity of the film is promoted by an increase in the amount of MoS_2_. It is observed that the films remarkably absorb UV light with long wavelengths, whereas composite films produce a low-transmission response to ultraviolet light in Region I. In Region II, all composite materials are highly translucent. This is because they have high transmittance percentages (they range between 85% and 96%) in the visible region. As expected, the ZnO film containing 2 mg of MoS_2_ exhibits a lighter color than the ZnO film containing 4 mg of MoS_2_. The undoped film has a lighter appearance. The lower transmittance observed for 4 mg MoS_2_ doping amounts can be attributed to the agglomeration of ZnO particles for higher amounts of MoS_2_.

### 2.2. Crystallographic Properties of ZnO/MoS_2_ Composite Films

X-ray diffraction (XRD) is a technique that reveals structural information, such as crystal structure, crystallite size, chemical composition, and strain. It can be used to analyze thin films and powders.

Since X-rays are waves of electromagnetic radiation, some of these waves cancel one another out in most directions, and some strengthen other waves in a few specific directions. This relationship is determined by Bragg’s law:

n λ = 2 **d** sinθ (3)

Here, **d** is the spacing between crystal planes, θ is the incident angle, n is an integer, and λ is the wavelength of X-rays [25].

To calculate the crystallite’s size, Debye–Scherrer’s equation is used [26]:

β_size_ = K λ/L cosθ (4)

where β_size_ is the peak width at half-maximum, K is the constant of the X-ray source, and L is the crystallite size.

The Debye–Scherrer formula only describes the effect of the crystallite’s size on the XRD peak’s broadening; it explains nothing about the lattice’s microstructures, i.e., the intrinsic strain that is formed in nanocrystals due to point defects, grain boundaries, triple junction, and stacking defects [27]. The broadening of the XRD peak occurs due to the size and microstrain of nanocrystals, and the total broadening can be written as follows.

β_hkl_ = β_size_ + β_strain_ (5)

Intrinsic strain affects the physical broadening of the XRD profile, and strain-induced peak broadening can be expressed as follows:

β_strain_ = 4ε tanθ (6)

where *ε* is the intrinsic strain of the material. The average particle size (crystallite size) and strain can be calculated using the Williamson–Hall equation [28]:

β_hkl_ = K λ/L cosθ + 4ε tanθ (7)

**Figure 7:**
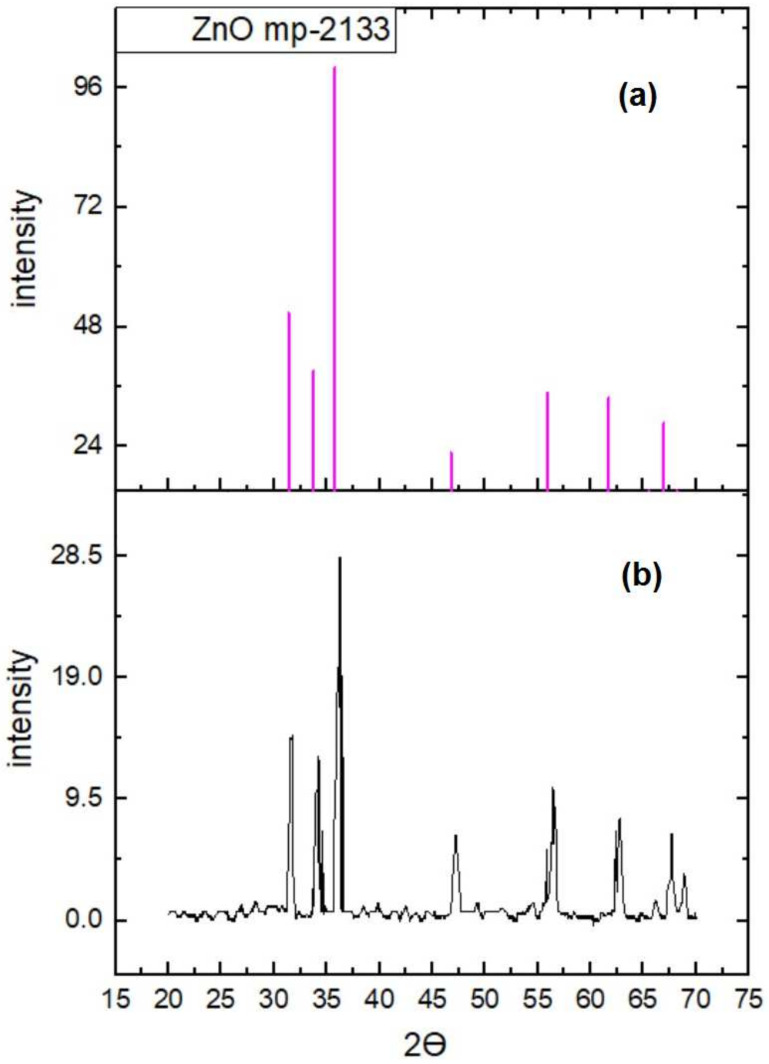
The XRD (**a**) standard and (**b**) experimental data of the transparent ZnO/MoS_2_ oxide film.

The XRD plot of the transparent ZnO/MoS_2_ oxide film with 4 mg of MoS_2_ content is shown in Figure 7b. It is observed that the transparent ZnO/MoS_2_ oxide film exhibits a hexagonal wurtzite structure (ICDD: 36-451), as shown in Figure 7a.

**Figure 8:**
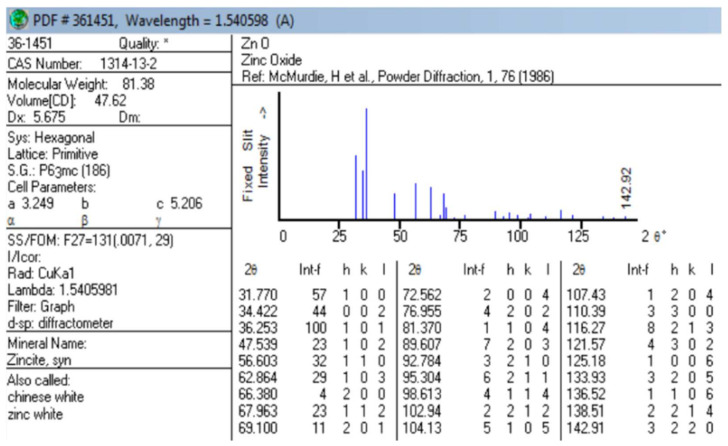
The ICDD standard of X-ray powder diffraction data relative to zinc oxide structure [29].

The XRD pattern is used for the identification of the crystalline phase and microstructural analyses of the prepared transparent ZnO/MoS_2_ oxide thin films. The pattern obtained has been indexed as a hexagonal unit cell with a wurtzite structure (ICDD Card No. 36-1451) [29], as shown in Figure 8. No evidence of MoS_2_ was found in the XRD response of the film. The reason for this is that the temperature applied to the film during the annealing process does not crystallize MoS_2_, and the amount of MoS_2_ is lower than the amount of ZnO. The Materials Project online platform was used to match the structure. Figure 7a shows the best coherent structure (mp-2133) for the material [30]. It can be observed in Figure 7b that films are polycrystalline. The observed relative peak intensities and interplanar spacing have been compared to that of their standard values, and they are shown in Table 2. Accordingly, the Miller indices of the crystal planes corresponding to 2θ angle values are shown in Table 2.

All peaks of the film correspond to the hexagonal wurtzite structure of ZnO, which has been studied by many researchers [31,32,33,34,35,36]. The XRD responses of the film corresponding to the (100), (002), and (101) planes are more evident. The indexed diffraction peaks were used to calculate interplanar spacings, and **d** corresponds to the planes of the hexagonal (wurtzite) ZnO crystalline structure via Bragg’s Law (Equation (3)). The crystallite sizes of all Miller indices were calculated from the Debye–Scherrer formula (Equation (4)). Table 2 presents the calculated crystallite size and interplanar spacing values of the ZnO/MoS_2_ film. The average crystallite size is calculated at about 23 nm. The highest relative error of **d** was calculated as 1.449%. In other words, since this error can easily be ignored, it shows that the zinc oxide structure predicted via the ICDD standard and the film prepared in this study have the same structure.

Rearranging the Williamson–Hall Equation, Equation (7) yields

**Figure 9:**
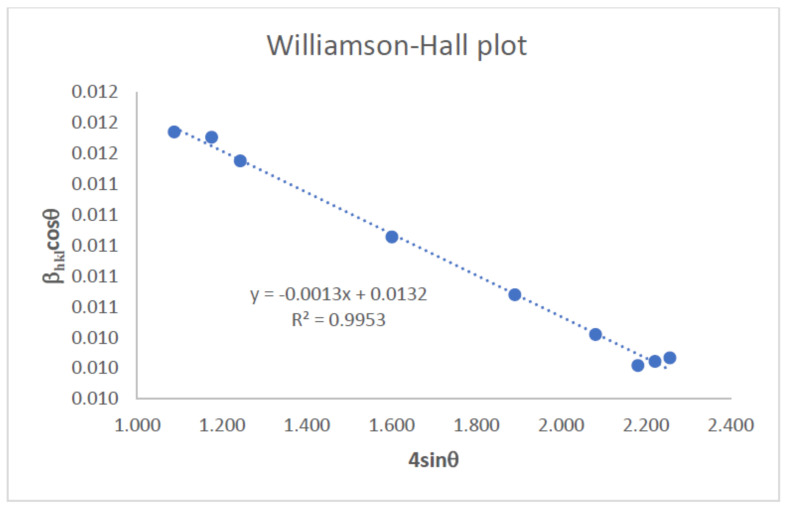
Williamson–Hall plot of transparent ZnO/MoS_2_ film.

β_hkl_ cosθ *=* K λ/L + 4ε sinθ (8)

which is an equation of a straight line. Equation (8) provides information about the isotropic nature of crystals. Figure 9 shows the plot of Equation (8), with the term (4sinθ) along the apsis and (β_hkl_ cosθ) along the ordinate axes corresponding to each diffraction peak of the ZnO/MoS_2_ film. This plotted straight line is a good-fitted line that corresponds to all values, as the correlation coefficient value of R^2^ is 0.9953. The slope of this straight line provides the value of the intrinsic strain, ε, whereas the intercept provides the average particle size of the ZnO/MoS_2_ film. The origin of the lattice strain is mainly attributed to lattice expansion or lattice contraction in the nanocrystals due to size confinement because the atomic arrangement is also slightly modified due to size confinement compared to their bulk counterparts. On the other hand, many defects are also created in the lattice structure due to size confinement, and this in turn results in lattice strain [27]. The negative slope of the fitted line in a Williamson–Hall plot indicates the presence of compressive strain in the crystal lattice of the specimen, while a positive slope indicates tensile strain [34]. The average particle size was determined approximately as 19 nm from this plot. From the slope, intrinsic strain ε was calculated as −0.0013. Since the slope is negative in Figure 9, the intrinsic strain comprises a compressive strain in the crystal lattice.

### 2.3. SEM, TEM/EDS, and AFM Measurements of ZnO/MoS_2_ Composite Film

**Figure 10:**
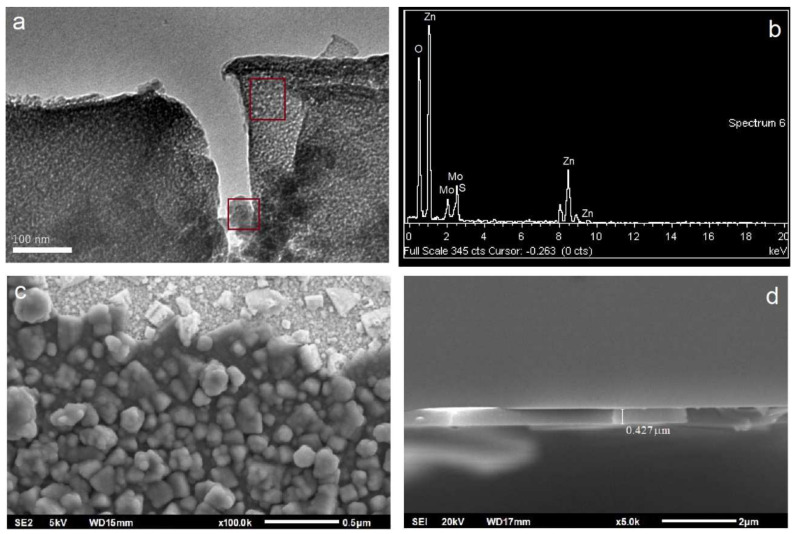
TEM image (**a**), EDS spectrum (**b**), SEM surface image (**c**), and SEM cross-sectional view image (**d**) of the ZnO/MoS_2_ composite film with 3 mg of MoS_2_. Red boxes in (**a**) show the regions selected for the elemental analysis.

HR-TEM analysis, as shown in Figure 10a, clearly revealed the formation of ZnO nanoparticles placed on MoS_2_ sheets. Also, Figure 10a shows several MoS_2_ layers surrounded by ZnO nanoparticles. The detailed results of EDS analysis in the regions marked with rectangular red frames are provided in Figure 10b. In this manner, the existence of ZnO nanoparticles and MoS_2_ sheets was proven. The surface is coated with compact ZnO nanoparticles (as observed in SEM surface examinations in Figure 10c) so that ZnO/MoS_2_ grow inhomogeneously. This observation is consistent with the homogeneity state determined via UV–vis spectroscopy and the crystal size determined via XRD. The results show the successful synthesis of ZnO/MoS_2_ structures. Finally, in the SEM cross-section image in Figure 10d, it can be observed that the thickness of the formed film was determined as 427 nm, which is consistent with the profilometer results in Table 3.

**Figure 11:**
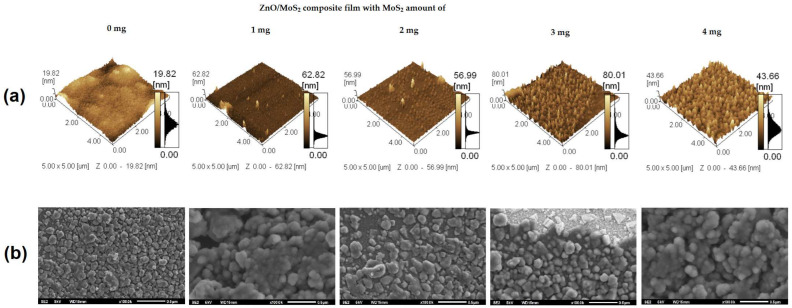
AFM images (**a**) and SEM images (**b**) of ZnO/MoS_2_ composite films with different amounts of MoS_2_.

With respect to the AFM characterization results shown in Figure 11a, the image on the left shows the formation of ZnO nanoparticles. With an increase in MoS_2_ content, it is observed that there is an increase in the frequency of peaks on the surface. For the addition of 4 mg of MoS_2_, the film’s surface is homogenous. Figure 11b presents the SEM images of the films, showing several MoS_2_ layers surrounded by ZnO nanoparticles. In line with the AFM results, SEM images show that the increment of MoS_2_ content in ZnO/MoS_2_ films leads to an increase in agglomeration; finally, for the addition of 4 mg of MoS_2_, the film’s surface becomes homogenous.

### 2.4. Sheet Resistance Measurements of ZnO/MoS_2_ Composite Films

**Figure 12:**
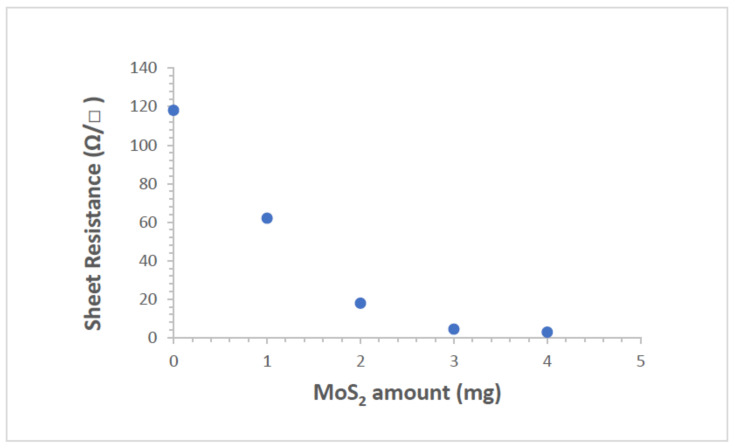
Sheet resistance of ZnO/MoS_2_ composite films with respect to MoS_2_ amounts.

To determine the sheet resistance of ZnO films with square geometry MoS_2_, the four-point probe method was used. The sheet resistance of ZnO films for different MoS_2_ amounts is shown in Figure 12. The sheet resistance of composite films decreased from 118.1 to 2.8 Ω/□ as the MoS_2_ amount increased from 0 to 4 mg. Additionally, the sheet resistance value is a very important parameter for the improvement of the electrical conductivity mechanism of ZnO/MoS_2_ composite films due to the generation of increased donor levels and an increase in free charge carriers. When MoS_2_ is doped with ZnO, Mo and S atoms merge into the ZnO lattice; then, free charge carriers increase in composite films. This change in structure contributes to conductivity, which decreases the sheet resistance of composite films.

Although the sheet resistance value is an important parameter, it does not make much sense on its own for transparent conductive films. For this reason, evaluating the transmittance (T) percentage of the film together with the sheet resistance (Rs) value is necessary. With Haacke’s definition (Equation (9)), these two parameters can be accurately calculated [37]. The figure-of-merit (FoM) value can be evaluated for the presence of an effective transparent conductor.

FoM = T^10^/Rs (9)

The transparent conductor characteristics of ZnO/MoS_2_ composite films are provided in Table 3. According to Table 4, the best TCO prepared via the sol–gel method with the highest FoM value is AgZnO (silver nanowire zinc oxide) [38]. Compared to the FoM values in Table 4, which covers previous studies on various transparent doped ZnO conductive oxides, it is possible to conclude that these new films have a structure that is very similar to the transparent conductivity characteristics of other films, and some are even superior relative to some MoS_2_ amounts. The thickness of each layer was measured at random locations, and their average was taken as the data point. The deposition mainly followed linear behavior, with a uniform increase in thickness between 22 and 24 nm for each deposited layer. The final thickness values after coating four more layers on the first film layer are provided in Table 3. For different films with different MoS_2_ amounts, film thickness varied within the range of 393 nm to 423 nm. Implementing measurements, it was shown that the ZnO/MoS_2_ composite film’s thickness did not change significantly according to the amount of MoS_2_. These composite films can doubtfully be used in all areas where transparent conductors are used.

## 3. Conclusions

In this study, ZnO/MoS_2_ composite films with various MoS_2_ amounts were fabricated via the sol–gel method in order to study the structural, optical, and electrical properties of composite films. The optical studies confirm a good dispersion of MoS_2_ in the ZnO film structure. The area under the curve and normalized width data verify the good performance of the fabricated composite films. The proper ultrasonication process was realized to maintain a good dispersion of the MoS_2_ inside the ZnO matrix, lowering the normalized width and increasing the resonance ratio. The best dispersion was obtained for the ZnO film with 4 mg of MoS_2_.

Structural investigations showed that the zinc oxide films are polycrystalline and have a wurtzite (hexagonal) structure. The intrinsic strain value was found to be 0.0013, which is a comparable result for zinc oxide composites. For future studies, it may be interesting to examine the mechanical properties by coating these films on a flexible substrate rather than a glass substrate. The transmittance of the films exhibited a range between 87 and 93% at a wavelength of 550 nm. The films are transparent in the visible region. This result allows for their application as transparent oxides. The film’s thickness varied within the range from 382 nm to 429 nm. Moreover, the sheet resistance values of composite films ranged from 118.1 to 2.8 Ω/□. The figure-of-merit values were in the order of 10^−2^, allowing for the use of composite films as transparent conductors. By using the four-point probe method, the ZnO/MoS_2_ film recorded low sheet resistance values relative to moving charges, which qualifies its use in photovoltaic applications. The findings demonstrate that one of the successful options for optoelectronic devices—which require large areas for application—may be the ZnO/MoS_2_ composites. This study provides a new approach for identifying dispersibility from optical properties and altered figure-of-merit values, which can be used in further optoelectronic and solar cell applications.

## 4. Materials and Methods

**Figure 13:**
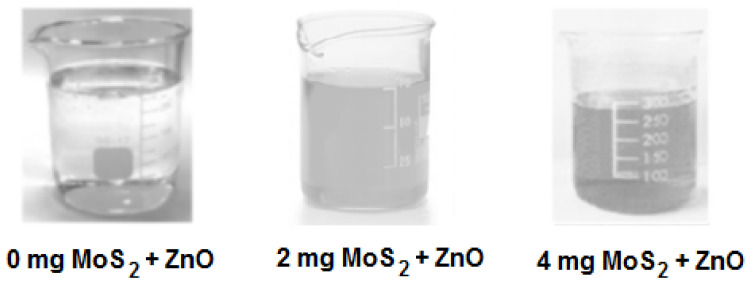
Photographs of ZnO solutions prepared with various MoS_2_ amounts in beakers.

**Figure 14:**
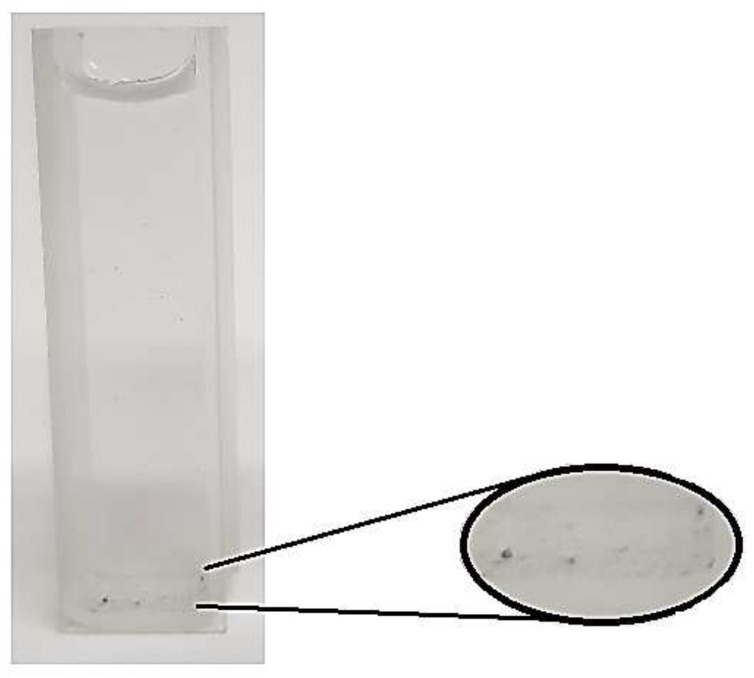
Photo of the ZnO solution with 1 mg of MoS_2_, prepared without ultrasonic treatment. **Inset:** MoS_2_ precipitation in the solution.

ZnO/MoS_2_ composite films were prepared using the sol–gel method. Zinc acetate dihydrate (ZnAc) was used as a precursor material in order to obtain ZnO’s structure. Diethanolamine (DEA), which is a surface-active material, was used to accelerate the dissolving procedure. Pure water (PW) was added for hydrolysis reactions. Sol was prepared by dissolving ZnAc in isopropanol (2propanol), applying magnetic stirring and using a hot plate set at 60 °C. After all precursor materials were dissolved, molybdenum disulfide (MoS_2_) was added to the solution. A homogeneous and stable sol was prepared. Then, DEA was added, and a vigorous stirring process was performed under ultrasonic conditions with a frequency of 40 kHz. After condensation, the precursor solution was hydrolyzed using ZnAc:2propanol:DEA:PW:MoS_2_ with a volume ratio of 0.4:3.5:0.2:0.25: (0, 1, 2, 3, 4 mg). Figure 13 illustrates ZnO solutions with MoS_2_ amounts of 0 mg, 2 mg, and 4 mg. As expected, the ZnO film containing 2 mg of MoS_2_ exhibited a lighter color than the ZnO film containing 4 mg of MoS_2_. The undoped film exhibited a lighter appearance. Hence, as the amount of MoS_2_ in the solution increased, the darker color indicated that MoS_2_ was well dispersed in ZnO. In addition, it is observed in Figure 14 that MoS_2_ in the solution precipitates when ultrasonic treatment is not applied while preparing the solution.

The photographs taken just after the gelation of solutions in the beakers (Figure 13) are in agreement with the composites exhibiting a high transmittance percentage (ranging between 85% and 96%) in the visible region (Figure 6). This result confirms the structural homogeneity in Figure 13, similarly to those shown in Figure 6. Moreover, the transmittance curves of the composites in Region II (Figure 6) are in agreement with the observations in Figure 13.

**Figure 15:**
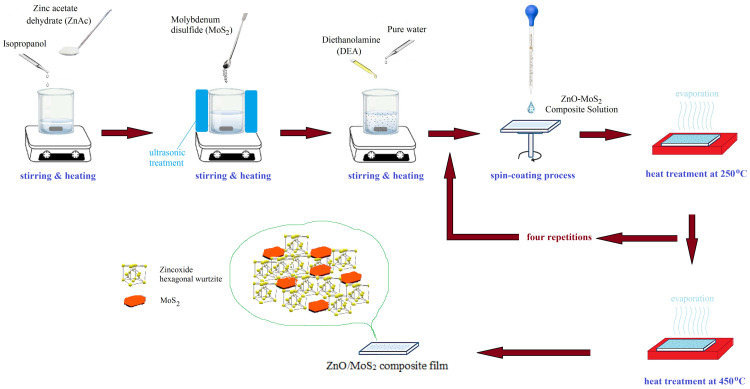
Preparation of coatings via the sol–gel method.

Corning 2947 glasses were cut with a diamond blade to obtain squares with sides measuring 1 cm. After all the cut glasses were washed with glass detergent and cleaned with ethanol, they were washed in an ultrasonic bath in order to clean the stains and then dried. The obtained solution was deposited on Corning 2947 glass substrates via spin-coating deposition (1000 rpm/30 s). After coating, ZnO/MoS_2_ composite films were immediately placed in a microprocessor-controlled (CWF 1100) furnace that had been preheated to 250 °C. The first layer on the substrate was formed. The coating and annealing processes were repeated four times to achieve the desired high conductivity of the film (Figure 15). The films were taken out of the furnace at the end of the last annealing process at 450 °C and left at room temperature.

The structures of composite films deposited on Corning 2947 substrates were characterized using an X-ray diffractometer (XRD, Philips PW-1800, Cu Kα radiation). The XRD spectra of films were recorded by scanning 2θ within the range of 20–50°. To match the XRD peaks with the proper Miller indices, the International Centre for Diffraction Data (ICDD) and Materials Project online platforms were used. UV–vis spectroscopic analyses of films were performed using a UV–visible absorption spectrophotometer (Perkin–Elmer Lambda 900 with Labsphere integrating software Spectrum 10) within the spectral range of 190–1100 nm wavelengths. The area under the curve and full width of the half-maximum of absorbance data calculations were performed using Origin 8.0 Software, focusing on absorbance data within the range of 190–400 nm wavelengths. To determine the sheet resistance of obtained films with square geometry, the four-point probe method was used. Furthermore, UV–vis spectroscopy was used to recognize the solution’s dispersion in order to improve dispersion scales, which are based on the reorganization of MoS_2_ dispersion in the solution. The background area underwent comparisons with the resonance ratio and normalized width. The thickness of the films was evaluated using a Stylus Profilometer (Veeco, Dektak 150). Stylus profilometer measurements were taken according to ISO 4287 and ISO 4288 standards. The surface morphology was characterized using a transmission electron microscope (HR-TEM), an atomic force microscope (AFM) in dynamic mode (Model SPM-9500, Shimadzu Corp. Kyoto, Japan), and a field emission scanning electron microscope (FESEM, Hitachi S4160, Tokyo, Japan).

## Figures and Tables

**Table 1:** The calculations of the resonance ratio and normalized width of transparent ZnO/MoS_2_ oxides.

Sample with Doping Amount of MoS_2_ in mg	Area of Resonant Band	Area of Nonresonant Background	Resonance Ratio	Width of Resonant Band (nm)	Height of Resonant Band (au)	Normalized Width (nm/au)
0	14.0664	2.7493	5.1164	113	0.1546	730.9185
1	90.7485	20.1200	4.5104	160	0.1714	933.2439
2	101.9256	21.6664	4.7043	160	0.2025	790.0064
3	116.8828	18.3885	6.3563	86	0.2250	382.1859
4	104.6548	10.0995	10.3624	50	0.2354	212.4104

**Table 2:** Calculations of the crystallites’ size and spacing between the planes of the XRD data of transparent ZnO/MoS_2_ oxides.

Peak Position	FWHM	Crystallite Size	Calculated from Bragg’s Law	ICDD Theoretical Value					
2θ (°)	β hkl (°)	L (nm)	d (Å)	hkl	2θ	d (Å)		4sinθ	β cos θ
31.556	0.699	21.545	2.835	100	31.405	2.848	0.455	1.088	0.012
34.186	0.702	21.609	2.623	002	33.780	2.653	1.139	1.176	0.012
36.222	0.696	21.897	2.480	101	35.777	2.510	1.200	1.243	0.012
47.192	0.691	22.880	1.926	102	46.790	1.942	0.831	1.601	0.011
56.433	0.694	23.688	1.630	110	55.909	1.645	0.884	1.891	0.011
62.724	0.699	24.276	1.481	103	61.729	1.503	1.449	2.082	0.010
66.105	0.698	24.756	1.413	200	65.543	1.424	0.743	2.182	0.010
67.493	0.706	24.692	1.388	112	66.941	1.398	0.737	2.222	0.010
68.707	0.713	24.637	1.366	201	68.173	1.376	0.719	2.257	0.010
		**Lavg = 23.331 nm**							

**Table 3:** Transparent conductor characteristics of ZnO/MoS_2_ composite films.

MoS_2_ Amount (mg)	Film Thickness (nm)	Sheet Resistance (Ω/□)	Transmittance (%) at 550 nm	Figure of Merit (FoM)
0	423 ± 2	118.112	93.464	4.31 × 10^−3^
1	393 ± 1	62.014	89.860	5.54 × 10^−3^
2	412 ± 4	17.857	88.715	1.69 × 10^−2^
3	423 ± 2	4.358	87.381	5.96 × 10^−2^
4	414 ± 3	2.842	86.907	8.65 × 10^−2^

**Table 4:** A review of various transparent doped ZnO conductive oxides with their sheet resistance and transmittance values.

Material	Deposition Method	Sheet Resistance (Ω/□)	Transmittance (%)	Figure of Merit (FoM)	Reference
AgZnO (silver nanowire zinc oxide)	Sol–gel	8	91	4.87 × 10^−2^	[38]
AZO (aluminum zinc oxide)	Sol–gel	148.43	66.23	1.09 × 10^−4^	[39]
Ga/ZnO (gallium zinc oxide)	PLD	40.6	90	8.59 × 10^−3^	[40]
In/ZnO (indium zinc oxide)	PLD	42.3	85	4.65 × 10^−3^	[40]
F/ZnO (iron zinc oxide)	PLD	24.15	90	1.44 × 10^−2^	[40]
Si/ZnO (silicon zinc oxide)	PLD	41.33	80	2.60 × 10^−3^	[40]
In/ZnO (indium zinc oxide)	Spray pyrolysis	32	90	1.09 × 10^−2^	[41]
Cl/ZnO (chlorine zinc oxide)	CVD	41.9	80	2.56 × 10^−3^	[42]
AgNW/GZO (silver nanowire gallium zinc oxide)	Atmospheric pressure plasma jet	68.3	7.3	3.03 × 10^−3^	[43]
rGO/ZnO (reduced graphene oxide zinc oxide)	Microwave-assisted sol–gel	80	3.24	3.31 × 10^−2^	[44]
CuO/ZnO (copper oxide zinc oxide)	Successive ionic layer adsorption and reaction (SILAR)	20	0.41	2.70 × 10^−7^	[45]
AIZO (aluminum indium zinc oxide)	Spray pyrolysis	85.8	25.5	8.48 × 10^−3^	[46]

## Data Availability

The data that support the findings of this study are available from the corresponding author upon reasonable request.
